# Two Biological Active Fractions Isolated from *Buthotus schach* (BS)Scorpion Venom Examined on Striated Muscle Preparation, In-vitro

**Published:** 2012

**Authors:** Hossein Vatanpour, Farhad Ahmadi, Abbas Zare Mirakabadi, Amir Jalali

**Affiliations:** a *Department of Toxicology and Pharmacology, School of Pharmacy, Shahid Beheshti University of Medical Sciences, Tehran, Iran.*; b *Razi Vaccine, Serum Production and Research Institute, Karaj, Iran.*; c *Department of Toxicology and Pharmacology , School of Pharmacy, Toxicology Research Center, Ahvaz Jundishapur University of Medical Sciences, Ahvaz, Iran.*; d *Student Research Committee, School of Pharmacy, Shahid Beheshti University of Medical Sciences, Tehran, Iran.*

**Keywords:** Twitch, Purification, Crude venom, Contracture, *Buthotus schach*

## Abstract

*Buthotus schach *is one of the most dangerous scorpions in tropical part of Iran. The effects of its crude venom at 1, 3, 10 μg/mL and its obtained fractions by gel filtrations were investigated on neuromuscular transmission. CBC and MHD indirectly and directly stimulated preparations techniques were used to study their possible pre or post junctional activities. At 3 and 10 μg/mL (not at 1 μg/mL), BS venom caused initiall increase in twitch height followed by blockage due to large contraction that responded gradually at the same time. Contracture responses to exogenous Ach (1-2 mM, 30 sec) and Carb (30-40 μM, 60 sec) in the presence of the venom were not increased which does show no anticholinstrease effects. Furthermore Contracture response to KCl (20-40 mM, 30 sec) does changed exposure to venom in CBC preparations. On the other hand the effects of the venom in response to directly stimulated preparations was shallower than in indirect stimulated preparations. So in agreement with KCL response BS venom affects mostly prejunctionally to facilitate the neurotransmitter release rather than postjunctionally effects. To access bioactive components, seven fractions were collected by gel filtrations techniques. Among the fractions F_6_, LD_50_=21 μg < F_4_, LD_50_= 35.5 μg < Venom LD_50_= 84 μg per mice were more toxic respectively. Both fractions show the same effects but stronger than venom on twitch height responses in indirectly stimulated CBC preparations.

Finally, according to our results venom as well as fractions F_4_ and F_6_ act mostly prejunctionally on Ach release. More attempt is carrying out to study their effects on ion channel activities.

## Introduction

Scorpion venoms are rich sources of peptides with the varieties of pharmacological functions cause massive discharge of catecholamines and death. Scorpion neurotoxins’s target are Na^+^ and K^+^ channels (1, 2). Among 1500 species of scorpions (3, 4) species Schach so called Hottentotta Zagrosensis (5) from Buthotus genous and Buthidae family is a dangerous scorpion in IRAN (1, 6, 7, 8).

Nirthanana, *et al., *has reported a marked reversible contracture in CBC nerve-muscle preparation caused by the black scorpion *Heterometrus spinifer *venom which was blocked by *d*-tubocurarine but not by tetrodotoxin (12). The results of previous studies confirm that scorpion venoms mostly have highly potent pre-synaptic activity rather than postsynaptical effects (12, 13).

The effects of several other scorpions, such as *Anderoctonous crasicuda, Mesobuthus epus, and Odontobuthus doriae *venoms and their fractions on neuromuscular transmission were reported previously in other works (9-11). Scorpion venoms can cause paralytic effects on nerve-muscle preparations either prejunctionally on transmitter release or postjountionaly on musle fibers.

In this study the effects of *Buthotus schach *scorpion venom as well as its fractions were studied on striated muscle using CBC and MHD indirectly and directly stimulated preparations.

## Experimental

Scorpion crude venom was gifted by Dr. Zare, Poisonous Animal Dep., Razi, Vaccine, Serum Production and Research Institute, Karaj-Iran and was reconstituted in 0.9% NaCl solution for use, when it was needed.

Mice and Chicks were purchase from animal unit in Pasture inistitute, Tehran-Iran. They were kept under standard conditions and fed with water and food according to the Guidelines for the use and care of Lab. Animals, published by the National Academy Press, which was accepted by the ethnic committee of the AUSR in Iran.

*Isolated chick biventer-cervices (CBC) nerve-muscle preparation*

Chicks aged 1-7 days were euthanized with CO_2_ and exsanguinated. The biventer cervices muscle with attach nerve was dissected and placed in an organ bath (5 mL) under 0.5 to 1 g of tension. Thyrode physiological solution with following composition (in mM) was used: NaCl, 118.4; KCl, 4.7; MgSO_4_, 1.2; KH_2_PO_4_, 1.2; NaHCO_3_, 25.0; Glucose, 11.1; CaCl_2_, 2.5, which was bubbled with O_2_ and maintained at 32ºC.

Supramaximal voltage 5-10 mV square-wave pulses of 0.2 ms duration for indirect and voltage 15-25 mV square-wave pulses of 2 ms duration for direct stimulation both at 0.1 Hz were used with a Narco trace physiograph and Bioscience stimulator. Contractions were measured using an isometric force transducer and preparation was allowed to equilibrate for 30 min.

Washout was then repeated until twitch tension returned to its original amplitude. Contractures to various exogenous agonists were subsequently recorded in the absence of electrical stimulation before, and after, incubation with venom. The final bath concentrations and periods of incubation were as follows:

Acetylcholine (Ach, 1 mM) for 30 sec; Carbachol (Carb, 30-40 μM) for 60 sec; potassium chloride (KCl, 20-40 mM) for 30 sec. Following addition of toxin, twitch tension amplitude was monitored for 4 h, or until twitches were abolished (14).

*Isolated mouse phrenic nerve-hemidiaphragm preparation*

Male mice (20-25 g) were killed by CO_2_ and decapitated. Hemidiaphragms and attached phrenic nerves were dissected as described by Bulbring (1946), it was mounted in 5 mL bath, containing physiological solution with above content (pH = 7.3) at 36ºC and gassed with O_2_.

For direct stimulation, tubocurarine (10 mM) was added to the organ bath to ensure that acetylcholine release from the nerve terminal did not contribute to the directly evoked twitch component in CBC and MHD preparations (15).

*Statistical analysis*

The twitch tension measurements were expressed as the mean ± standard error of the mean (SEM) (n = 4). Differences between groups or treatments were compared using Student t-test, with p < 0.05 indicating significance.

*Purification*

Fractioning of the soluble venom was accomplished using a Sephadex G50 column equilibrated and eluted with a pH = 8.3, 0.1 M ammonium acetate buffer (16). Protein content was estimated spectrophotometrically with Bradford method at 595 nm (17). Fractions were concentrated, lyophilized and kept at room temperature.

As a toxicologic index LD_50_ were determined in mice (balb-c, 20 g) through the IV injections according Reed and Muench method (18, 19).

**Figure 1:**
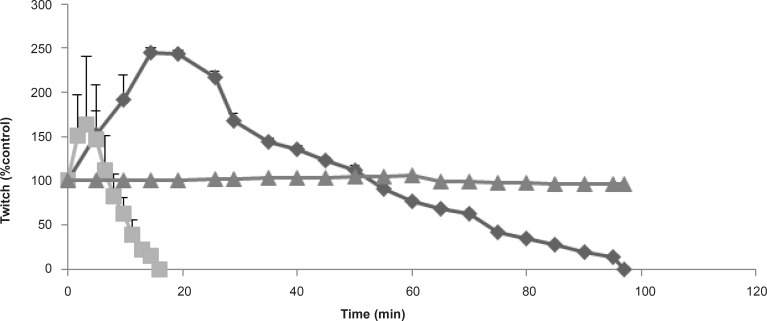
The effect of *Buthotus schach *scorpion venom at 1 μg/mL (▲), 3 μg/mL (♦); 10 μg/mL (■) concentrations, in response to indirect CBC stimulated preparations. Each point represents the maximum response for that concentration (mean ± SEM; n = 4).

## Results and Descussion

*Effects of the venom on CBC preparations*

*Buthotus schach *scorpion venom at 1, 3, 10 μg/mL concentrations were examined in both indirect and direct muscle stimulation in a time and concentration-dependent manner. At 3, 10 μg/mL of the venom twitch height was increased and followed by reduction in responses to indirect stimulation of the Chick Biventer Cervicis nerve-muscle preparations. The effect of 10 μg/mL venom was extremely stronger and due to large contracture, twitch height showed a lower transient increase compared with 3 μg/mL. However *Buthotus schach *venom at 1 μg/mL did not cause significant effects neither on twitch height nor on contracture responses (Figure1).

**Figure 2:**
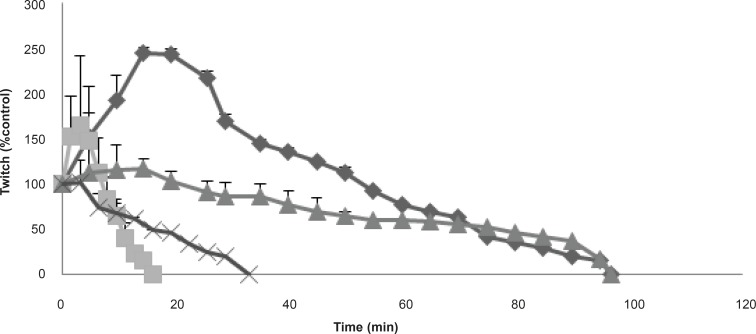
The effects of the venom at 3 μg/mL on twitch height in response to directly (▲) stimulated CBC preparations in compare with indirectly (♦) stimulated preparations are shown. Furthermore, its effects at 10 μg/mL are also shown on twitch height at both directly (●) and indirectly (■) CBC stimulated preparations. Each point represents the maximum response for that concentration (mean ± SEM; n = 4).

In order to identify the effects of the venom on pre- or postjunctionally, the effects of the venom at 3 and 10 μg/mL was also tested on twitch height responses in CBC directly stimulated preparations (in the presence of tubocurarine). At both concentrations, the effects of the venom on twitch height in responses to direct stimulations was not as potent as responses to indirectly stimulated preparations (Figure 2).

**Figure 3:**
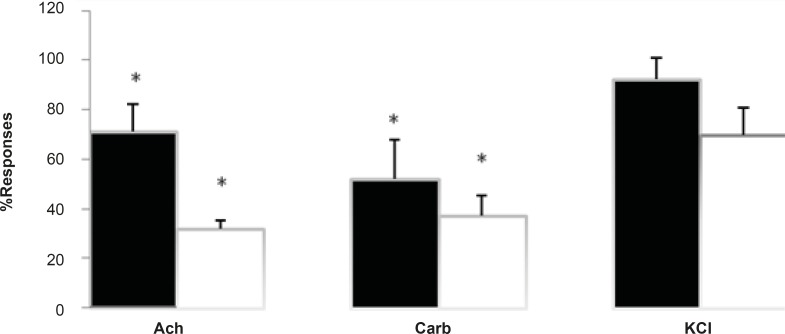
The effect of *Buthotus schach *scorpion venom at 3 μg/mL (■) and 10 μg/mL (□) concentrations in response to exogenous Ach (1-2 mM) for 30 sec, Carb (30-40 μM.) for 60 sec and KCl (20-40 mM) for 30 sec in CBC preparations. Each column represents the maximum response (% control) for that concentration. (Mean ± SEM; n = 4) (* = p < 0.05).

Furthermore, due to an increase in twitch height, the effects of the venom was tested to investigate for possible anticholinesterase activities as well as direct mucle paralysis. Responses to exogenous Ach, Carb, and KCl were examined with exposure to *Buthotus schach *venom in CBC nerve-muscle preparations in the absence of stimulations. Results show a significant (p < 0.05) decrease in response to Ach (1-2 mM, 30 sec) and Carb (30-40 μM, 60 sec). However it showed no significant changes in responses to KCl (30-40 mM, 30 sec) in CBC preparations (Figure 3).

*Effects of the venom on MHD preparations*

**Figure 4:**
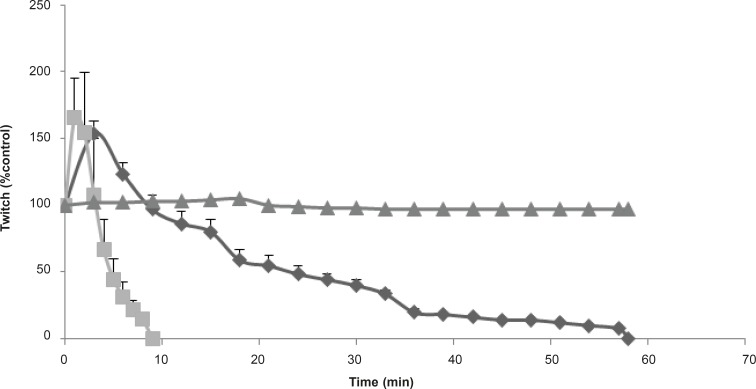
The effect of *Buthotus schach *scorpion venom at 1 μg/mL (▲), 3 μg/mL (♦); 10 μg/mL (■) concentrations, in response to indirect stimulation of MHD preparations. Each point represents the maximum response for that concentration (mean ± SEM; n = 4).

In order to investigate more prejunctional effects, *Buthotus schach *scorpion venom at different concentrations (1, 3, 10 μg/mL) were tested in indirectly stimulated MHD preparations. At 3 and 10 μg/mL, venom initially caused a transient increase in twitch height followed by neuromuscular paralysis (Figure 4). The effect of 10 μg/mL venom was extremely stronger and blockage occurred within 10 min.

*Fractionation of the venom by gel filtration*

**Figure 5:**
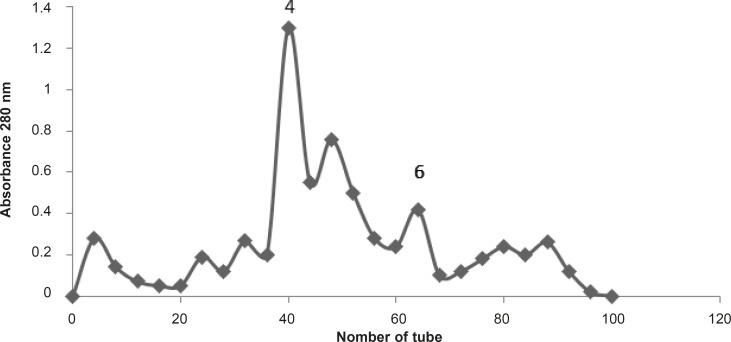
*Buthotus schach *scorpion venom (300 mg) was dissolved in Ammonium acetate buffer and loaded on Sephadex G50 column with 1 mL/min flow rate. Seven fractions were obtained. F_4_ and F_6_ are two pharmacologically active and responsible for the venom effects

To achieve the bioactive molecules responsible for pharmacological effects of the BS scorpion venom, it was fractionated by Gel filtration technique using G50 column and ammonium acetate buffer with 1 mL/min flow rate (Figure 5). 

**Figure 6:**
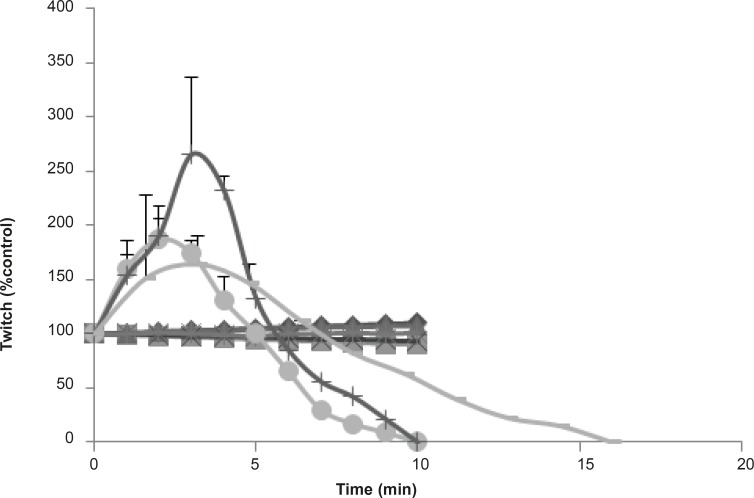
The effects of *Buthotus schach *scorpion venom and its seven collected fractions were examined each at 10 μg/mL concentrations on CBC indirectly stimulated nerve–muscle preparations. Fractions F_1_ (♦), F_2_ (■), F_3_ (▲), F_5_ (**×**), F_7_ (_*_), had shown no effects on twitch height or muscle contractions responses. However fractions F_4_ () and F_6_ (**+**) were shown a transient increase in twitch height followed by huge contracture led to muscle paralysis similar to one that was seen with the venom (●).

Subsequently, to follow the same pharmacological experiments as crude venom, attempt were made for seven collected fractions to see if there is any affects similar to venom. Each fraction was used at 10 μg/mL concentrations on chick biventer cervicis indirectly stimulated nerve-muscle preparations (Figure 6).

Moreover, two fractions called F4 and F6 showed a transient increase in twitch height followed by huge contracture led to muscle paralysis similar to the venom. The toxicity of both pharmacological effective fractions in comparison with the venom were determined in mice (balb-c, 20 g) according to Reed and Muench method. Our results show their toxicity as follows: (F6, LD_50_ = 21 μg/mice < F4, LD_50_ = 35.5 μg/mice < Crude venom, LD_50_ = 84 μg/mice).

Protein assay for F_4_ and F_6_ fractions was 26% and 13% of the total amount of the venom respectively.

## Discussion

Scorpion venoms mostly consist of neurotoxins (20) which can cause several physiological disturbances in human body leading to death. *Buthotus schach *scorpion from tropical area of IRAN is a dangerous scorpion which takes many victims through the sting, everyday. *Buthotus schach *scorpion venom as well as its fractions were studied on neuromuscular junction using CBC and MHD preparations. Others have reported several toxins from different scorpions all over the world that can interact with ion channels as well as neuromuscular transmission in vitro (21-24).

The effects of *Buthotus schach *scorpion venom on indirect nerve-muscle stimulated preparations caused a rapid initial increase in twitch height followed by slow contractions that eventually inhibited both twitch and contractions responses in CBC preparations. Its effects on MHD twitch height in response to indirectly stimulated preparations confirm its effects mostly to increase Ach release from nerve ending.

As these effects may also contribute to anticholinestrase activities, the effect of the BS scorpion venom was tested on exogenous Ach, Carb responses in the absence of stimulation of CBC nerve-muscle preparations. Due to no increase in response to exogenous Ach and Carb there are no anticholinsterase activities. This effect was also similar to Odontobuthus doriae scorpion venom effect (11).

Further to our findings regarding the Buthotus schach scorpion venom on transmitter release, its effects on direct stimulated muscle preparations exposure to d- tubocurarine showed no significant effects. This was in contrast with other scorpion venom effects on muscle fibers and their contactility (9, 10, 25, 26).

To seek for the active biomolecules, responsible for the venom effects, two active fractions (F_4_ and F_6_) were isolated and found out that their effects on CBC indirectly stimulated preparation were similar to venom effects even stronger. Their toxicity in comparison with the venom showed F_6_ as its effects on CBC indirect stimulated preparations is the most toxic fraction of the venom on neuromuscular junctions.

**Table 1:** LD50 and other pharmacological results of the venom and its active fractions (F_4_ and F_6_) on indirectly stimulated CBC nerve-muscle preparations

	**LD**_50_ ** μg/mice**	**Max. Twitch height in response to indirect stimulation (% ctl)**			**Time to block NMJ (min)**		
		**1μg/mL**	**3μg/mL**	**10μg/mL**	**1μg/mL**	**3μg/mL**	**10μg/mL**
**Crude venom**	84	106	244	164	_	97	16
**F**_4_	35/5	105	247	187	_	20	10
**F**_6_	21	105	217	265	_	20	10

Finally, *Buthotus schach *scorpion venom predominantly attributed prejunctionally to increase the Ach release from nerve ending in neuromuscular junctions. However F_6_ possible peptide isolated fraction has stronger effects than crude venom in these aspects. More investigations are required to find out the real mechnisam action of the venom as well as its fraction. 
